# Gene Saturation: An Approach to Assess Exploration Stage of Gene Interaction Networks

**DOI:** 10.1038/s41598-019-41539-w

**Published:** 2019-03-21

**Authors:** Ziqiao Yin, Binghui Guo, Zhilong Mi, Jiahui Li, Zhiming Zheng

**Affiliations:** 1 Beijing Advanced Innovation Center for Big Data and Brain Computing, Beihang University, Beijing, 100191 China; 2 Shenyuan Honors College and School of Mathematics and Systems Science, Beihang University, Beijing, 100191 China; 3 LMIB and Peng Cheng Laboratory, Shenzhen, 518055 Guangdong China

## Abstract

The gene interaction network is one of the most important biological networks and has been studied by many researchers. The gene interaction network provides information about whether the genes in the network can cause or heal diseases. As gene-gene interaction relations are constantly explored, gene interaction networks are evolving. To describe how much a gene has been studied, an approach based on a logistic model for each gene called gene saturation has been proposed, which in most cases, satisfies non-decreasing, correlation and robustness principles. The average saturation of a group of genes can be used to assess the network constructed by these genes. Saturation reflects the distance between known gene interaction networks and the real gene interaction network in a cell. Furthermore, the saturation values of 546 disease gene networks that belong to 15 categories of diseases have been calculated. The disease gene networks’ saturation for cancer is significantly higher than that of all other diseases, which means that the disease gene networks’ structure for cancer has been more deeply studied than other disease. Gene saturation provides guidance for selecting an experimental subject gene, which may have a large number of unknown interactions.

## Introduction

More than 60,000 genes have been identified in the NCBI database (https://www.ncbi.nlm.nih.gov/), of which more than 20,000 encode proteins. Most of these genes do not work independently but are regulated by each other to achieve a variety of complex biological processes. The human genome project successfully mapped the human genome in 2003, and with many subsequent studies, our understanding of individual genes has improved. However, our research into the relationships between genes remains in its infancy. The first study on gene interactions recorded by NCBI was published in 1967^1^. There are molecular interactions between genes in a cell that connect genes into networks called *Real Regulatory Networks* (RRN). However, such intermolecular interactions cannot be directly and extensively explored but are only hypothetical based on macro data of possible intermolecular interactions.

There are more than 2.5 million publications on genes in PubMed. In total, 35508 of them are on gene interactions within *Homo sapiens*, which covers 289,946 interaction relations. These gene interaction data are collected from 5 external sources: BIND (http://www.bind.ca), BioGRID (http://thebiogrid.org/), EcoCyc (http://www.ecocyc.org), HIV-1 protein interactions (https://www.ncbi.nlm.nih.gov/RefSeq/HIVInteractions/, provided by: Southern Research Institute) and HPRD (http://www.hprd.org). Gene-gene interaction relations contain physical interactions, such as two-hybrid and genetic interactions, including negative/positive genetic interactions. The gene-gene interaction network contains more types of interaction relations, such as Affinity Capture-RNA, than traditional Protein-Protein Interaction networks (PPI).

In the early stage of gene network research, biological experimental data were insufficient for researchers to build a gene network. When only minimal information about the relationship between genes is available, the network built based on such information is divided into many small parts. Many features of the RRN cannot be identified in this case. Hence, to recreate the RRN as closely as possible, many researchers have obtained impressive results by constructing gene networks by taking the conclusions of multiple biological studies into account^2–6^. A method for building gene networks based on the literature was proposed in 2001^2^. With the development of machine learning, methods that use text mining to build gene networks have been accepted^4,5^. However, most of the relations in such gene networks have not been experimentally demonstrated, which may make our original data noisy.

Recently, with the development of high-throughput biotechnology, gene interaction networks can be built based on experiment results rather than text mining^7^. There are many types of molecular biological networks: Protein-Protein Interaction networks (PPIs)^8,9^, Coexpression Networks^10–12^, Disease Gene Networks (DGNs)^13^, Gene Functional Networks^7,14,15^, Pathway Gene Networks^16,17^, and Gene Interaction Networks^18,19^. However, these networks are still not RRN. Some of them are subnetworks of the real regulatory network, while others include different relations between genes. We refer to these networks the *Measured Regulatory Network* (MRN). As gene-gene interaction relations are constantly being explored, gene networks are also constantly changing^20,21^. There is no doubt that results will be influenced by such incomplete and noisy biological data.

**Figure 1:**
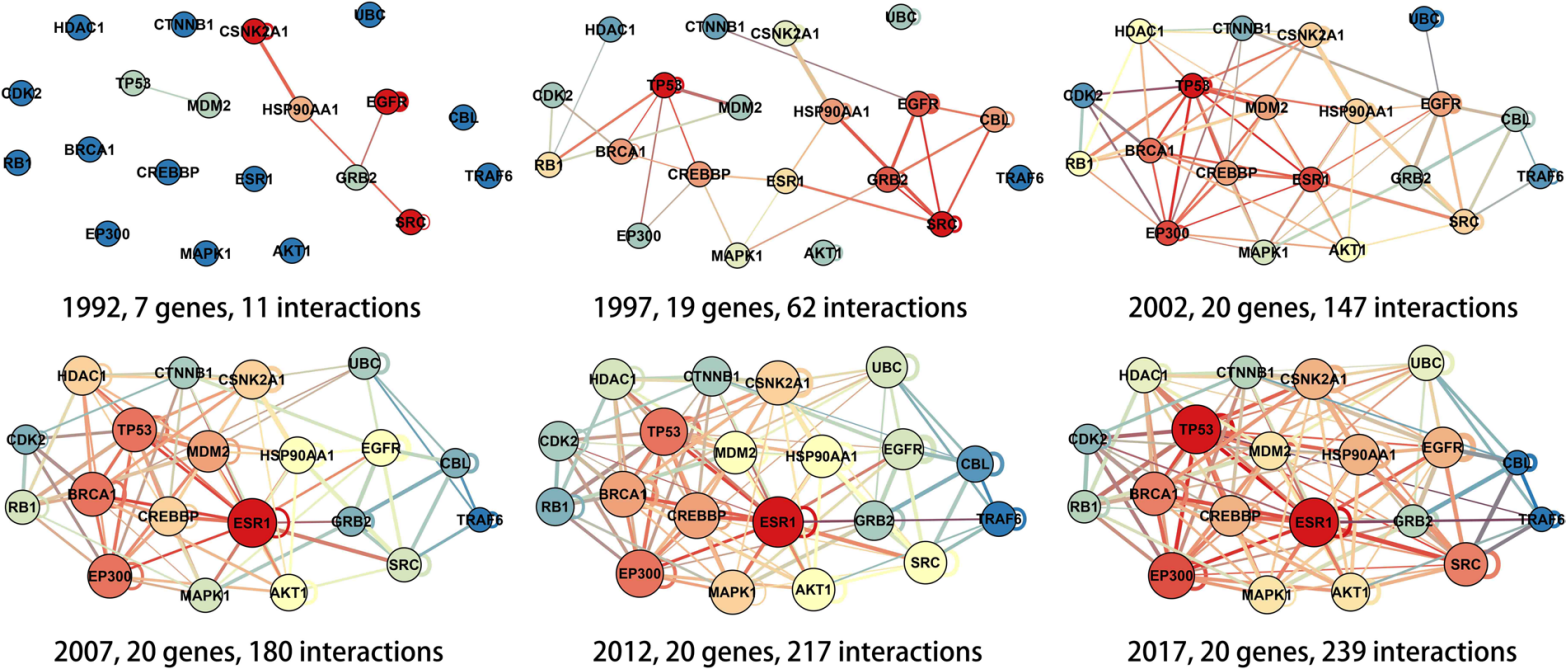
Networks of the 20 top genes of the number of related references. Changes in the interaction network of the 20 top genes of related references from 1992 to 2017. The time interval between the two adjacent pictures is 5 years.

The changes in gene networks based on results from newly published studies should not be ignored. Figure 1 shows the different interaction networks of the same set of genes (the 20 top genes with the largest number of related studies) at different times. Only 7 of these 20 genes interacted 25 years ago. Five years later, 19 had interactions, and the number of interactions increased from 11 to 62 in 1998. Every 5 years thereafter, more than 20 interactions were found between these 20 genes. Therefore, if the studied object network contains some of these genes, the results could be completely different 25 years ago versus now. Moreover, the interactions have not been fully described; thus, the studied object network for researchers will be MRN but not RRN, which will last for a long time.

**Figure 2:**
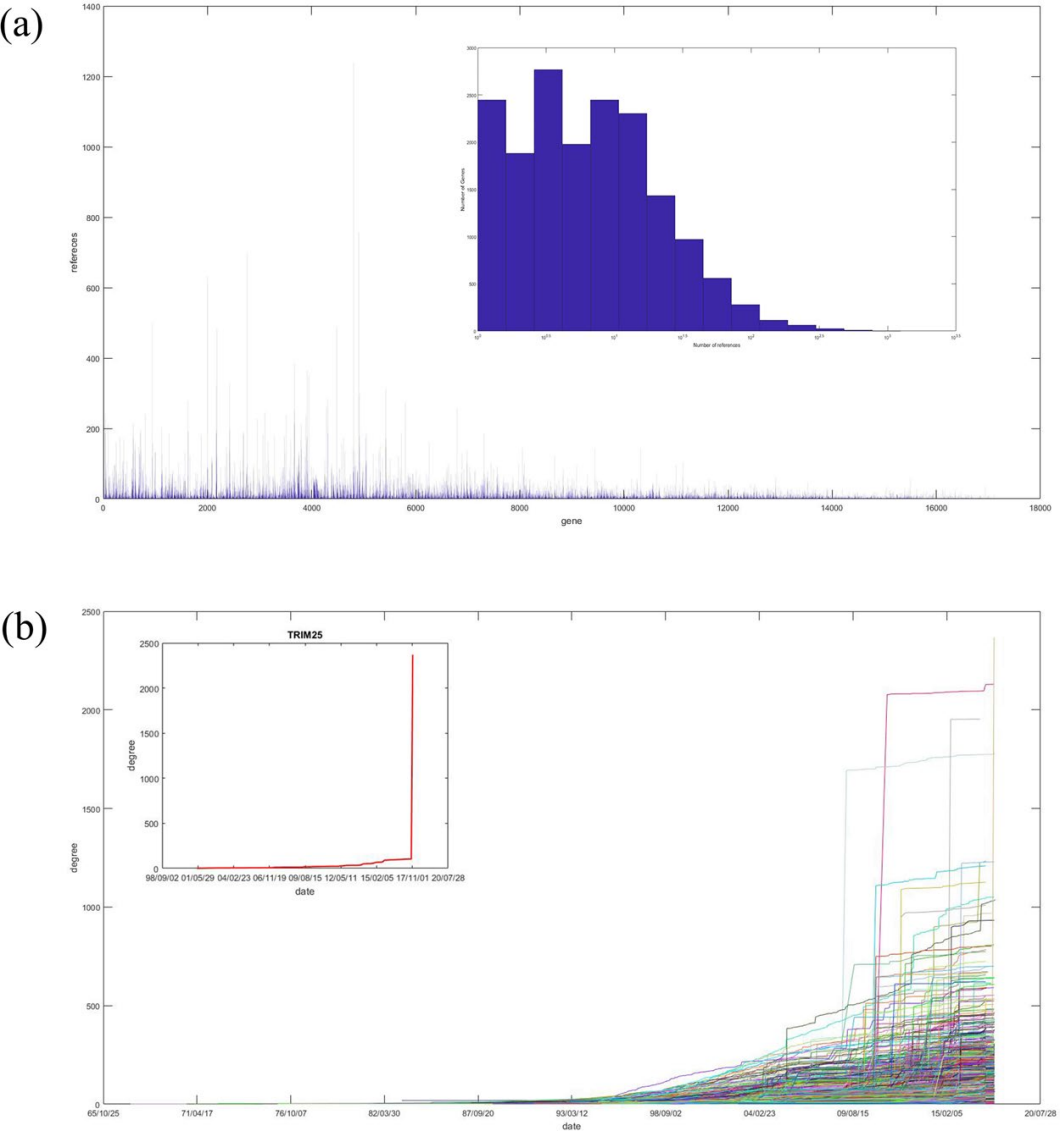
(**a**) The relationship between the number of gene related studies and the degree of each gene in gcHGIN. The red line shows *n* = *k*, and the green line shows *n* = *ak*, where *a* is the average ratio of *n*_*i*_/*k*_*i*_ of all the genes in gcHGIN. The value of a here is approximately 0.4244. (**b**) Radian distribution of all 17274 genes in gcHGIN. (**c**) Degree distribution of all genes with a ratio *n*_*i*_/*k*_*i*_ = 1. The figure on the top right shows the increase in degree over time (DT increase curve) of the gene with the largest degree, MYOD1, among all these genes. (**d**) Degree-literature ratio versus date curve of the 20 top genes of the number of related studies.

There are numerous studies on the interactions between genes, but the numbers of studies on each gene are not evenly distributed. Here, the concept of network science must be introduced, namely, the giant component. If there is a path accessible between any two points in a subnetwork of the network, we call this subnetwork a component, and the largest component in the network is the giant component. In the giant component, each node has at least one connected node. The connection number of a gene is called the degree of this gene in network science. Figure 2a shows the relationship between the degree of gene *k*_*i*_ and the number of studies on this gene’s interaction *n*_*i*_ with all genes in *the giant component of human gene interaction network* (gcHGIN) based on data from NCBI, and there are 17274 genes with 289913 interactions based on the 2018-03-04 updated version. However, among the genes in gcHGIN, the genes with the most references are not those with the highest degrees. However, there are still problems. New edges will always be added to the network with the exploration of new interactions. For example, there are two studies on the yeast cell cycle network^14,15^. However, the research object of the study from 2008^15^ is not exactly the same as the study from 2004^14^.

**Figure 3:**
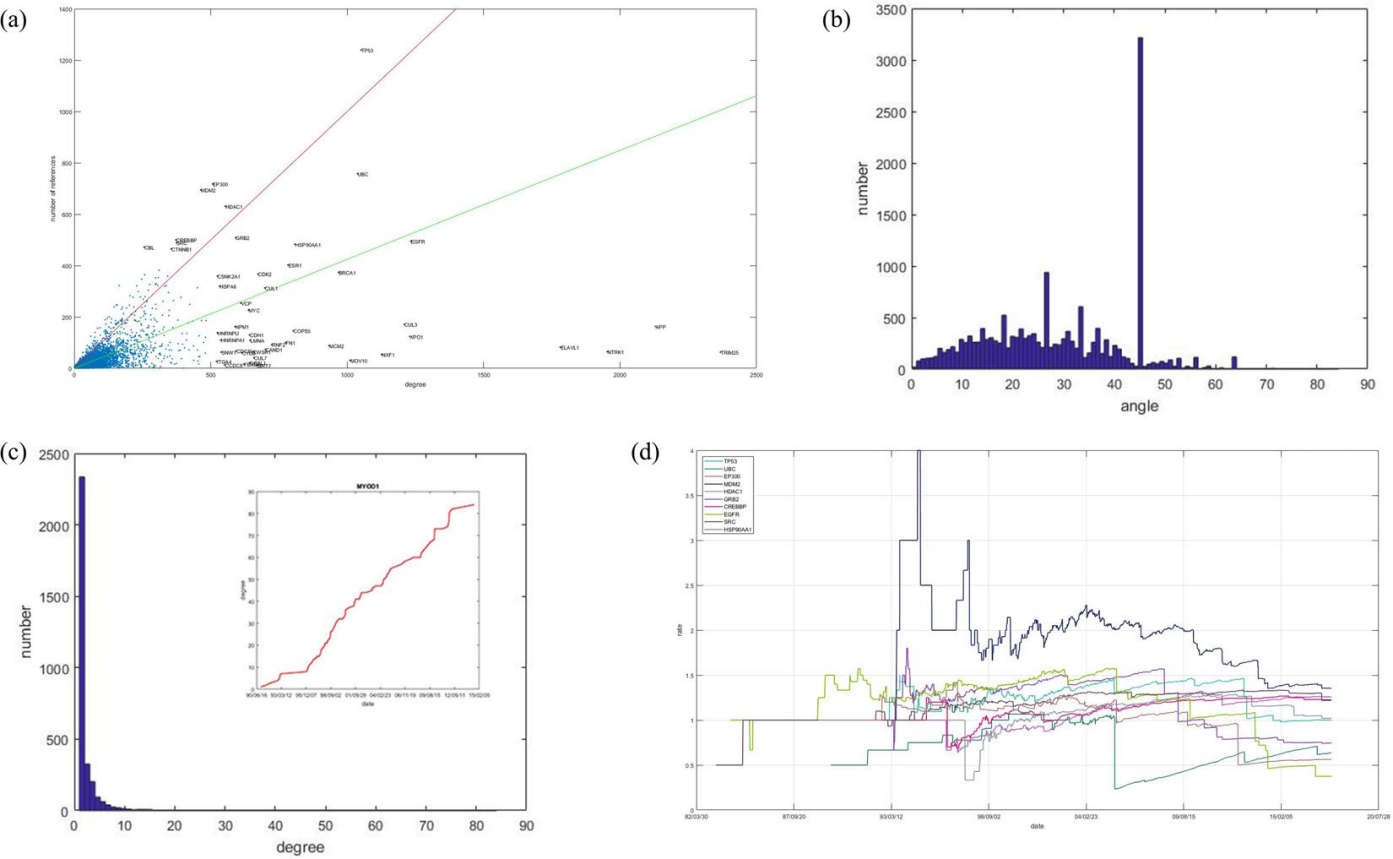
(**a**) Number of studies of all the genes in gcHGIN. The figure on the top right shows the distribution of studies of all these genes. (**b**) DT increase curve of all the genes in gcHGIN. The figure on the top left presents a special example, TRIM25, for which almost all of its interaction data are obtained from one study^22^.

Many disease genes are highly connected in the interaction network as essential genes, such as the cancer-related gene TP53, which has a degree of 1051 based on interaction data. As these highly connected genes are often the focus of research, the question of whether these genes are an area of focus given their wider function or simply because these genes have been intensively studied arises. Meanwhile, the related references for each gene are not evenly distributed. As shown in Fig. 3a, most genes have less than 10 related references, but there are some genes with more than 1000 related references. Further, the existence of this phenomenon may cause us to ignore some key genes that have not been widely studied, which may introduce mistakes in results based on incomplete and noisy data. Although we cannot obtain the RRN in the short term, we can establish an evaluation standard based on data to evaluate the accuracy and credibility of existing studies if a method is available that can estimate the distance between MRN and RRN.

## Results

### Gene interaction network

There are 2,459,379 records of gene interactions in 166 species in NCBI. Among them, 289,946 interactions are noted in *Homo sapiens* and involve 17309 genes. A gene-gene interaction network is built based on these data. Nodes represent genes and are connected to each other if there is an interaction record between them. The giant component of this gene-gene interaction network, *the giant component of human gene interaction network* (gcHGIN), is constructed with 17274 genes. The average degree of the nodes in gcHGIN is 33.37. The gene with the largest degree is TRIM25, whose degree is 2368. There are 1778 genes with degrees of 1 in the network.

The term “interaction” includes the direct physical binding of two proteins as well as their coexistence in a stable complex and genetic interaction (https://wiki.thebiogrid.org/doku.php/experimental systems). There may be more than one relationship between two genes. Some of the relations still cannot be described very well, such as an unspecified isoform of STAT3 that binds APRE^22^. Here, in our gene-gene interaction network, two genes are connected if there is at least one interaction between them. There will be no more than one edge between two genes even if there is more than one type of interaction between them.

Figure 1 shows the changing process in the network of the top 20 genes from the number of related references from 1992 to 2017. Only 7 of these 20 genes had reported interaction relationships in 1992. However, in 2017, all of these 20 genes had been proven to have more than 1 interaction partner. In addition, with the increasing knowledge of the network, the key genes are also changing. One of the most important genes in the network from 2017, estrogen receptor 1 (ESR1), is not included in the network from 1992. Thus, results based on the network consisting of these genes would be different if studies were performed at different times. Moreover, these 20 genes are the genes with the top related literature numbers. Regarding genes without such a focus, there may be more unknown relations that can significantly affect our results.

There are more than 2.5 million studies on genes in PubMed. In total, 35508 of these studies are on gene interactions within Homo sapiens that cover 289,946 interaction relations. Published information from all 35508 studies about gene interactions of the 17274 genes in gcHGIN is collected in PubMed (https://www.ncbi.nlm.nih.gov/pubmed) from May 23, 1967^1^, to Dec 2, 2017^23,24^. Figure 3a shows the distribution of the interaction-related number of studies of each gene in gcHGIN. The average related number of studies of all genes in gcHGIN is 14.16. More than half of the genes in gcHGIN have less than 10 interaction-related studies. Moreover, 2446 genes have only 1 interaction-related study. Meanwhile, there are only 262 genes with greater than 100 interaction-related studies, which accounts for less than 1.6% of all genes in gcHGIN. The gene with the largest number of published reports is tumor protein p53 (TP53), with 1239 studies. Thus, most of the studies focus on only a few specific genes, while the remaining genes have been rarely studied.

### Index formed by study numbers and degrees of genes

Since a gene’s degree in RRN is the upper limit of this gene’s degree, the number of studies is a continuously increasing number. Thus, gene saturation can be measured by *n*_*i*_/*k*_*i*_, where *n*_*i*_ is the number of studies on this gene’s interaction and *k*_*i*_ is the gene’s degree. In Fig. 2a, the radian between the data points of each gene and the X-axis, *arctan*(*n*_*i*_/*k*_*i*_), indicates the average contribution of the gene literature to the increase in the gene’s degree. Figure 2b shows the distribution of these radians. There is an interval with an extremely high number. *π*/4 is included in this interval. If a gene has a radian value of *π*/4, the gene has the same number of degrees and studies. Further, the degree distribution of all genes with a radian of *π*/4 is plotted in Fig. 2c. A large number of these genes are genes with only one study and with a degree of 1, which are the major contributors to the large number of genes with a radian of *π*/4. However, there are also some genes with larger degrees. The figure on the top right of Fig. 2c shows the curve of the increase of the degree over time (DT increase curve) of the gene with the largest degree among all these genes, MYOD1, which has a degree and literature number of 84. However, not all genes’ DT increase curves stably increase like MYOD1. Figure 3b shows the DT increase curve of all the genes in gcHGIN. The DT increase curves have different patterns. Some curves, such as that of MYOD1 in Fig. 2c, exhibit a steady increase, whereas others, such as that of TRIM25 in Fig. 3b, exhibit a sudden increase. For TRIM25, there is one key study^25^ that increases the gene’s degree from 105 to 2368. However, if the saturation of this type of gene is measured only with *n*_*i*_/*k*_*i*_, completely different results may be obtained before and after the key study was published.

A well-designed gene saturation index should be non-decreasing over time. Figure 2d shows the degree-literature ratio versus time curve of the 20 top genes based on the number of related studies. There are many decreasing intervals, which indicates that *n*_*i*_/*k*_*i*_ is not a well-designed gene saturation index. For example, there is a sudden decrease of UBC’s curve from 1.024 to 0.229 on Oct 1, 2005. Thus, more factors need to be considered to obtain a reasonable gene saturation index. The curve of the increasing of degree over time (DT curve) needs to be considered and indicates whether a gene’s degree is significantly increasing over time. If there is a curve that can fit the DT curve well, the feature of the increasing of degree over time can be obtained. This fit curve is called the fDT curve.

### DT curves and fDT curves

The degrees of genes increase with time. The DT curves of all 17274 genes in gcHGIN based on NCBI interaction data are shown in Fig. 3b. Each increase in the figure represents at least one published article on the interaction relationship of this gene. The value of the increase is the number of new interactions of the gene found in the article. The curves in the figure are discrete as the increases are based on the publication of articles. Although the first study on gene interactions collected by the NCBI database was published in 1967, the increase in the degree of genes before 2000 was very slow and more rapid after 2000.

Even if the recent degree and increasing rate are different for each curve, most curves have similar features that slowly increase at first, then suddenly rapidly increase, and finally slowly increase. This feature can be modeled by the *Logistic Growth Model*. The classical Verhulst logistic growth model was proposed in 1838^26^. There are numerous variations of the classical Verhulst logistic growth model. A. Tsoularis and J. Wallace reviewed and compared several such models and analyzed the properties of interest for them in 2002^27^. The classical Verhulst logistic growth model is designed to model intraspecific population dynamics and more general biological growth. The main feature of this model is that it increases slowly at first, then suddenly rapidly increases, and finally slowly increases. There are two key indexes in the classical model: carrying capacity *K* and the rate of natural increase *r*. The details are presented in the Method section. In the classical growth model, *K* represents the carrying capacity that the population will ultimately reach. Here, *K* represents the predicted interaction numbers in the RRN of a specific gene based on existing data. The other index *r* represents the natural increasing rate of the population without the limit of all the factors in the classical growth model. Here, *r* represents the maximum research focus concentration for a specific gene. With a larger *r* value, a gene’s degree has a higher probability to suddenly increase in a short time.

**Figure 4:**
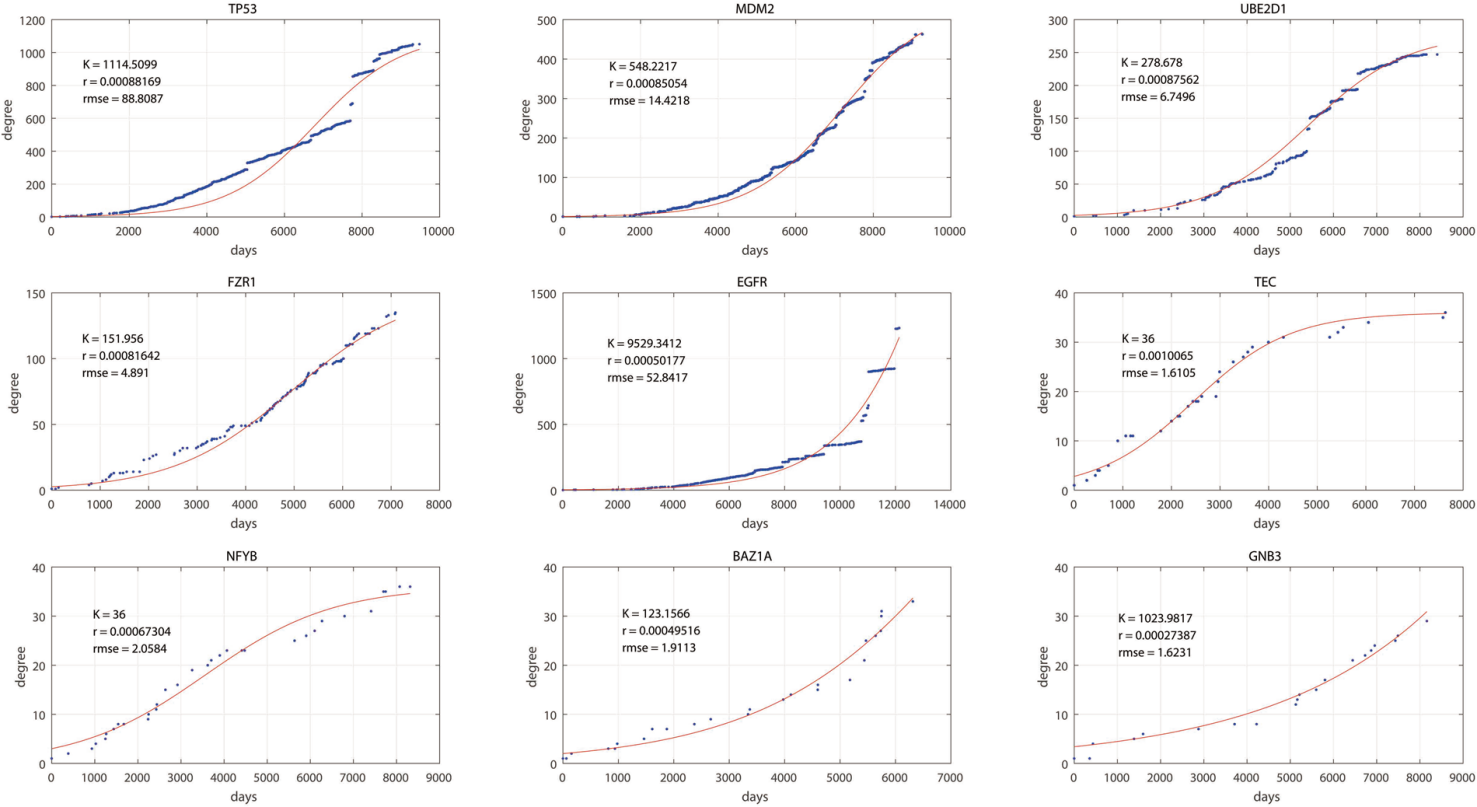
DT curve and fDT curve of some example genes. Nine example genes are presented with their DT curves and fDT curves. The blue dots in each figure represent the fDT curve, and the red line is the corresponding fDT curve. The values of the fit parameters K and r and the root mean squared error are plotted on the top left.

Figure 4 shows 9 example genes with their DT and fDT curves. The blue dots in each figure are the fDT curve, and the red line is the corresponding fDT curve. The values of the fit parameters K and r and the root mean squared error are plotted on the top left. When the number of related studies increases, the root mean squared error increases, which is similar to TP53’s fDT curve. The genes’ fDT curves still remain the main feature of their DT curves. These 9 genes’ fDT curves stop at different stages of the logistic growth function to date, which indicates that the studies on these genes are at different stages. The fit parameter K represents the predicted interaction numbers in the RRN, which is the upper limit of a gene’s degree. As one of the classical Verhulst logistic growth model’s features, the degree’s increasing speed continuously increases when the degree of the gene is less than *K*/2. However, when a gene’s degree is larger than *K*/2, the increasing speed of the gene’s degree slows. When this gene’s degree is close to *K*, the increasing speed of this gene’s degree is close to 0. This feature can be used to divide all these genes into three stages: the early stage, middle stage, and final stage. The genes shown in Fig. 4 represent these three types of genes. EGFR, BAZ1A and GNB3 are at their early stage. TP53, MDM2 and FZR1 are at their middle stage. UBE2D1, TEC and NFYB are at their final stage.

### Research focus concentration *r*

**Figure 5:**
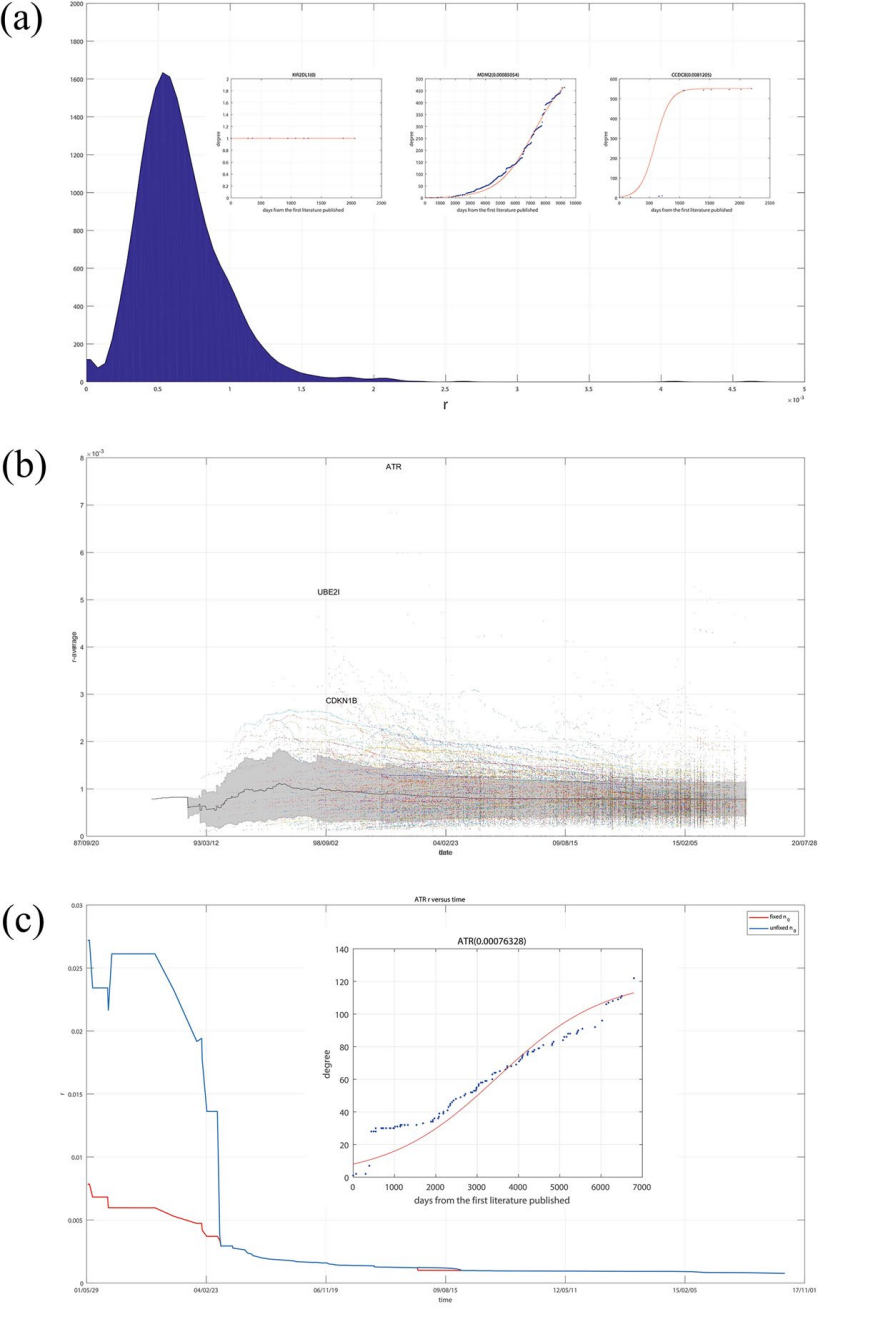
(**a**) Distribution of *r* for all the genes in gcHGIN using 3 example genes’ DT and fDT curves with *r* values that range from small to large. (**b**) Distribution of all the genes’ *r* values versus time. The black line is the average *r* of all the genes in gcHGIN, and the grey area is the area of the average value plus or minus one times the standard deviation. Points with different colors represent *r* values of different genes, and points with the same color represent *r* values of the same gene different times. (**c**) DT curve, fDT curve and the *r* value over time for the ATR gene, which exhibits the widest range of *r* values among all the genes in gcHGIN.

Figure 5a shows the distribution of r for all the genes in gcHGIN. Three example genes’ DT curves and fDT curves are presented in Fig. 5a and demonstrate that a larger r indicates that the gene’s degree rapidly increases over a short time, while a smaller *r* indicates that the gene’s degree slowly increases. Most genes’ *r* values are approximately 0.6 × 10^−3^. The gene with the highest *r* value (approximately 0.0081) is CCDC8, whereas the gene with the lowest *r* value (0) is KIR2DL1. There are significant differences between these two genes’ DT increase curves. CCDC8’s degree is 551 with 13 studies, and KIR2DL1’s degree is 1 with 10 studies. Although these two genes have similar numbers of reports, their increasing degree features are completely different. SMARCAD1’s degree increased rapidly when the first three studies were published and then slowed. KIR2DL1’s degree was 1 when the first study was published and has been maintained at 1 as subsequent studies have been published. The remaining degrees of genes’ increasing features involve these two genes.

As an index to describe the increasing feature of a specific gene, *r* should be stable over time. Figure 5b shows the distribution of all genes’ *r* values versus time. The black line is the average *r* of all the genes in gcHGIN, and the grey area is the area of the average value plus or minus one times the standard deviation. Points with different colors represent *r* values of different genes, and points with same color represent *r* values of same gene at different times. Although the average *r* value is unstable over the first few years, the average *r* value is stable at approximately 0.8×10^−3^. This stability is mainly attributed to the fact that Electrophoretic Mobility Shift Assay (EMSA) was previously used to study gene-gene interactions of human in the 1990s before human genome microarrays were commercially available^28–30^. With human genome microarrays, ChIP-on-chip technology can be used to study human gene-gene interactions, allowing the identification of the cistrome, which is the sum of binding sites for DNA-binding proteins on a genome-wide basis. As a result, there is only a limited amount of research on human gene interactions before 2000, which makes the average of the gene’s *r* value extremely unstable. Given the increasing amount of research published after the time when ChIP-on-chip technology became available, the average *r* value is increasingly stable.

For each gene, *r* should be stable. Figure 5b presents the only 3 genes that have *r* value values greater than twice the average value. These three genes have a similar feature that their degrees all exhibit a sudden increase at the beginning of the DT curve. Figure 5c shows the DT curve, fDT curve and *r* value over time for the ATR gene, which has the widest range of *r* values among all the genes in gcHGIN. The DT curve exhibits a sudden increase from 7 to 28 between two studies published in 1999^31,32^ and then remains stable around 30 for a long time, subsequently representing a normal Logistic Mode. The other two genes, UBE2I and CDKN1B, exhibit similar features. In Fig. 5c, there are three types of fDT curves of ATR. The three types of fDT curves have different *k*_0_ values, which include the degree when the first study was published, the average degree when 5 studies were published and unlimited k0. The fDT curve in yellow, with an unlimited *k*_0_, fits the DT curve best, but an unlimited *k*_0_ would make the range of *r* become wider than that of a fixed *k*_0_. However, the curve for which *k*_0_ equals the degree when the first study was published exhibits the worst fitting of the DT curve. Although the genes that exhibit a sudden increase at the beginning of their DT curves are minimal, this situation must be considered. As a result, the average degree when 5 studies were published has been chosen as the initial value of *k*_0_ that can fit the DT curve better but does not make *r* become unstable at the beginning stage.

### Principles of the gene saturation index

**Figure 6:**
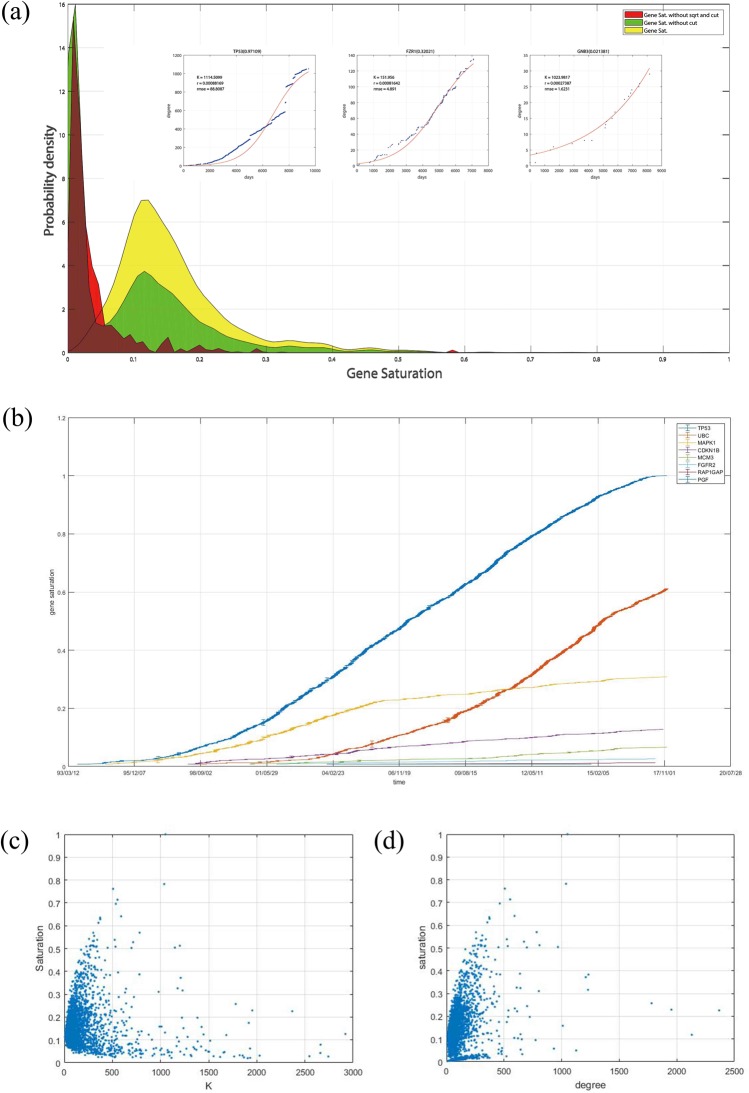
(**a**) Distribution of three types of gene saturations with three example genes’ DT curves and fDT curves with different gene saturations. (**b**) Saturation value versus time of 8 random genes. (**c**) Correlation between a gene’s degree and saturation. (**d**) Correlation between a gene’s predicted interaction numbers in the RRN *K* and at saturation.
- **Robustness principle**. The gene saturation index should be stable and should not suddenly increase over a short period of time. Gene saturation is an index that describes how much we have learned about a specific gene. In general, we cannot learn too much about a gene from one study. Indeed, we cannot deny that there are some remarkable studies that found a large amount of interaction partners for a single gene in one study. For example, Choudhury, N. R. *et al*. found more than 2000 interaction partners of TRIM25 in one study^25^. However, two points should be noted about this concept: a) these studies do not frequently occur; b) their occurrence may indicate that these genes have much more potential unknown interaction partners than others, i.e., they may have a higher degree in RRN compared with other genes.

Given that the gene saturation is an index that describes a gene’s local distance from MRN to RRN and that MRN approaches RRN over time, the gene saturation index should follow such principles:

- **Non-decreasing principle**. The gene saturation index should non-decreasing function over time. Gene saturation is an index that indicates whether we understand a specific gene’s interaction relationship very well. As each gene’s interaction partners are discovered, we increasingly understand the gene, and our knowledge is not reduced. Thus, the gene saturation index should non-decreasing over time.
- **Correlation principle**. The gene saturation index should have a strong correlation with the gene’s local distance from MRN to RRN but a very weak correlation with the gene’s degree in RRN. Although we cannot prove it, we believe that each gene’s partner numbers cannot be the same in the RRN. As shown in Fig. 6a, CDKN1B and FGFR2 both currently have a degree of approximately 90, but CDKN1B has considerably more interaction-related studies than FGFR2. The curve trends show that CDKN1B’s degree is more difficult to increase than FGFR2. Thus, although CDKN1B and FGFR2 have similar degrees, we can easily tell that CDKN1B’s gene saturation should be higher than that of FGFR2.

There are two factors that must be considered: the number of related studies and the number of unknown interactions of a gene. The data regarding the number of related studies can be found in the NCBI interaction data. The number of unknown interactions for a specific gene cannot be directly obtained but can be predicted from the logistic growth model mentioned above. The following formula can be used to describe gene *i*’s saturation ${G}_{s}^{i}$:

$${G}_{s}^{i}=\sqrt{\frac{{n}_{i}}{N}\cdot \frac{{k}_{i}}{{K}_{i}}},$$

where *n*_*i*_ represents the number of interaction-related literature numbers of gene *i*, *k*_*i*_ represents the current degree of gene *i*, *N* represents the standard number of gene interaction studies, and *K*_*i*_ represents the predicted interaction numbers in the RRN of a specific gene *i* by the method mentioned above. To ensure that the saturation index value is between 0 and 1, the value of *N* is set equal to the maximum number of related studies (1239), which is the number of studies on TP53’s interactions.

For the non-decreasing principle, as the variables and parameters of the definition of gene *i*’s saturation ${G}_{s}^{i}$, *n*_*i*_ and *k*_*i*_ are both non-decreasing variables, and *N* is a constant for all genes. If the predicted interaction numbers in the RRN of a specific gene *i* do not significantly change over time, a gene’s saturation value should be non-decreasing. Figure 6b shows 8 genes’ saturation values and increases at different times. Compared with the radian in the plane formed by the number of related studies and the degree of genes presented in Fig. 2d, the genes’ saturation values are more stable and rarely suddenly decrease.

For the correlation principle, Fig. 6c,d show the relationships among degree, *K* and gene saturation. As degree represents the degree of the gene in MRN and *K* represents the degree of the gene in RRN, the gene’s saturation has a normal correlation with the degree and a very weak correlation with *K*. Thus, regardless of a gene’s interaction partners in the RRN, its saturation could either be large or small because a gene with numerous interaction partners in RRN makes it easy to identify some of its interaction partners. However, it is difficult to identify all of the interactions. However, for a gene with a few interaction partners in RRN, it is possible to identify all of its interaction partners in a short period of time, but more studies are needed to prove the results. However, a gene with a higher degree is more likely to have higher gene saturation because a high degree always indicates that numerous publications on this gene are available.

Regarding the robustness principle, as genes’ related literature numbers and DT increase curves change with time, a gene’s saturation will be different when calculated using data from a different period. A gene’s saturation steadily grows. There is only one possible reason for a gene’s saturation to significantly increase over a short period of time, namely, numerous interaction partners of this gene were found in a short period of time. However, once such a case occurs, it may indicate that more unknown interaction partners exist. As a result, this type of gene’s saturation should not increase too much. Thus, in short, the gene saturation index should be stable, and all genes’ saturation values should not suddenly increase in a short period of time.

### Gene saturation and increasing rate of the gene degree

To evaluate whether a gene saturation index is well designed, the correlation coefficient between the gene saturation and the increase in the gene degree in the near future can be calculate. Interaction data before Jan. 1, 2016, are used to calculate the gene saturation at 2016. The increasing rate of the degree of these genes over the next two years from Jan. 1, 2016, to Dec. 2, 2017, was calculated. A strong negative correlation was noted between gene saturation and the increasing rate of the gene degree. Thus, a gene with higher gene saturation indicates a reduced increasing rate of the gene degree in the future that is closer to the value in the RRN.

**Figure 7:**
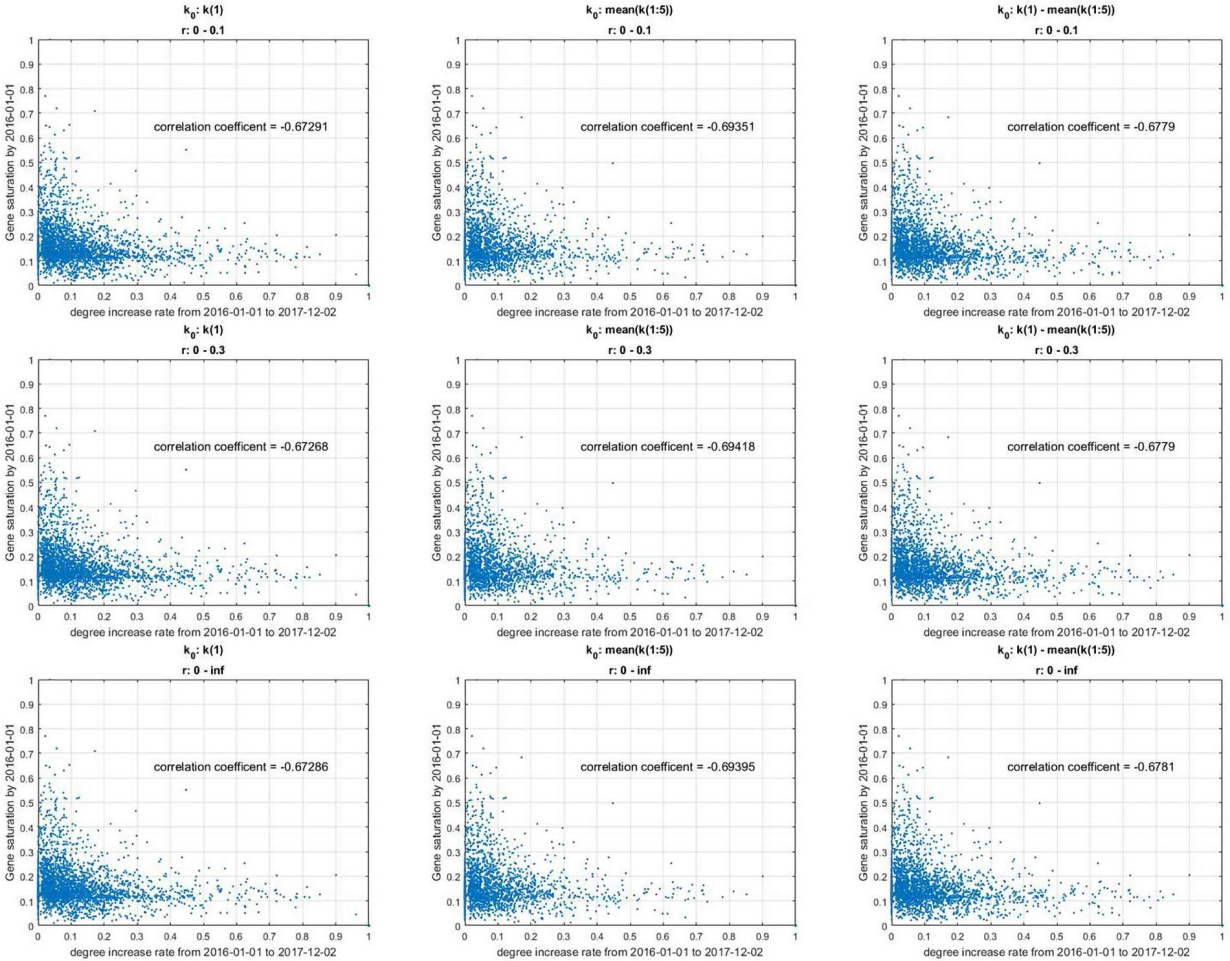
Correlation between gene saturation and the increasing degree in the near future with different ranges of fit parameters. The gene saturation value is calculated using data reported before 2016-01-01. The correlation coefficient is calculated based on gene saturation in 2016 and the increasing degree from 2016-01-01 to 2018-03-04. Each row represents results with the same range of *r* but a different initial value of *k*_0_, and each column presents results with the same initial value of *k*_0_ but a different range of *r*.

The range of parameters in the logistic growth model will not significantly affect this correlation coefficient. There are three parameters in the logistic growth model: real degree *K*, rate of natural increase *r* and initial degree *k*_0_. The correlation coefficients in different ranges of parameters are calculated, and the results are presented in Fig. 7. When different ranges of parameters are chosen in the model, the fDT curves are different, which may affect the gene saturation value. Figure 7 shows the correlation between gene saturation and the increase in the degree in the near future with different ranges of fit parameters. The gene saturation value is calculated using data before 2016-01-01. The correlation coefficient is calculated with the gene saturation at 2016, and the degree increases from 2016-01-01 to 2018-03-04. The numbers in each row are results with the same range of *r* but different initial *k*_0_ values, and the numbers in each column are results with the same initial k0 value but different ranges of *r*. All the results presented in Fig. 7 have a strong correlation with a correlation coefficient of approximately 0.7, which indicates that the gene saturation value can reflect the possibility of a gene’s unknown interaction partners being found in the near future and is rarely affected by the range of parameters.

In contrast to the results presented above, the correlation coefficient will be significantly smaller when the *K* value of the gene saturation value K is fixed at this boundary, which may be caused by the remarkable development of experimental techniques that have been used to study gene-gene interactions in the 2000s. Electrophoretic Mobility Shift Assays (EMSA) were used to study gene-gene interactions in humans before 2007, when human genome microarray became commercially available^28–30^. With the human genome microarray, ChIP-on-chip technology can be used to study human gene-gene interactions, which allows the identification of the cistrome, the sum of binding sites, for DNA-binding proteins on a genome-wide basis. As a result, the degrees of genes have begun to increase faster than ever noted before 2007; thus, the degrees of some genes without any study before this time point will increase very fast at the beginning stage. When these data are used to calculate the fDT curves of these genes, the fDT curve easily has a particularly large value at the final stage. Thus, the value of K would be fixed at this boundary. These fDT curves cannot predict the degree of genes in the RRN very well because these types of genes always share a similar feature: they exhibit a large degree but a small number of related studies. As a result, the correlation coefficient between gene saturation and the increasing rate of the gene degree will be significantly decreased. Thus, the gene saturation value of this part of the gene must be deleted to ensure the correlation coefficient between gene saturation and the increasing rate of the gene degree.

### Top genes in different orders

Table 1 shows the top 10 genes with highest gene saturation, and the gene saturation values of genes with the top 10 degree values and top 10 related literature numbers. Genes with the top related literature numbers are similar to genes with the top gene saturation values but differ from genes with the top degrees. These results mean that although gene saturation is calculated using both a gene’s related literature number and degree, a gene saturation’s value mainly depends on the number of related studies, which is similar to our expected results. In addition, when a gene has a high degree but a few related publications, this gene’s saturation is always very small or it does not have a saturation value because this kind of gene always has an unpredictable potential.

**Table 1:** Top genes sorted by saturation, literature number and degree.

ID	Symbol	Saturation	Sat. Rank	Literatures	Degree	Description
**Sorted by Saturation**						
7157	TP53	0.9711	1	1239	1051	Tumor protein p53
7316	UBC	0.7621	2	758	1038	Ubiquitin C
2033	EP300	0.7612	3	718	507	E1A binding protein p300
3065	HDAC1	0.7136	4	631	553	Histone deacetylase 1
4193	MDM2	0.6878	5	694	463	MDM2 proto-oncogene
2885	GRB2	0.6409	6	509	591	Growth factor receptor bound protein 2
1387	CREBBP	0.6353	7	500	372	CREB binding protein
6714	SRC	0.6282	8	489	374	SRC proto-oncogene, non-receptor tyrosine kinase
1499	CTNNB1	0.6126	9	465	355	Catenin beta 1
2099	ESR1	0.5696	10	402	783	Estrogen receptor 1
**Sorted by Literature Numbers**						
7157	TP53	0.9711	1	1239	1051	Tumor protein p53
7316	UBC	0.7621	2	758	1038	Ubiquitin C
2033	EP300	0.7612	3	718	507	E1A binding protein p300
4193	MDM2	0.6878	5	694	463	MDM2 proto-oncogene
3065	HDAC1	0.7136	4	631	553	Histone deacetylase 1
2885	GRB2	0.6409	6	509	591	Growth factor receptor bound protein 2
1387	CREBBP	0.6353	7	500	372	CREB binding protein
1956	EGFR	—	*	495	1233	Epidermal growth factor receptor
6714	SRC	0.6282	8	489	374	SRC proto-oncogene, non-receptor tyrosine kinase
3320	HSP90AA1	0.4604	21	483	808	Heat shock protein 90 alpha family class A member 1
**Sorted by Degree**						
7706	TRIM25	—	*	63	2368	Tripartite motif containing 25
351	APP	—	*	163	2130	Amyloid beta precursor protein
4914	NTRK1	0.2290	324	65	1953	Neurotrophic receptor tyrosine kinase 1
1994	ELAVL1	0.2573	242	82	1781	ELAV like RNA binding protein 1
1956	EGFR	—	*	495	1233	Epidermal growth factor receptor
7514	XPO1	0.3164	153	124	1229	Exportin 1
8452	CUL3	0.3715	89	171	1209	Cullin 3
10482	NXF1	—	*	54	1126	Nuclear RNA export factor 1
7157	TP53	0.9711	1	1239	1051	Tumor protein p53
7316	UBC	0.7621	2	758	1038	Ubiquitin C

Some of the genes in the table are denoted by ‘−’ in the Saturation column and ‘*’ in the Sat. Rank Column, which indicate that these gene’s *K* are fixed beyond the limited region. As a result, deletion of the saturation value may be unreasonable. For details, see the Methods section.

In the table of genes with the top 10 number of related studies, 8 of the genes are also in the top 10 for gene saturation values. There is only one gene, EGFR, in the top 10 genes with the highest number of related studies without a gene saturation value. EFGR’s DT and fDT curves are presented in Fig. 4. Although numerous related studies on EFGR’s interactions were previously published, studies on EFGR remain at their early stage. EFGR’s degree increased from approximately 400 to 1300 in the last three years, which makes us unable to tell whether all of EFGR’s interactions have been identified. These types of genes require further study to discuss whether more unknown interactions exist. In other words, the situations of these types of genes remain unstable, so more time is needed to confirm these findings. As a result, these types of genes do not have a gene saturation value, which indicates that the situation of these types of genes remains unstable.

In the table of genes with the top 10 degrees, 4 do not have a gene saturation value, and all of them except TP53 and UBC do not have top 10 gene saturation values. The gene with the top degree is TRIM25, which is mentioned above in Fig. 3b. Although TRIM25’s degree is 2368, most of these interactions are reported in only one study published in 2017^25^. However, before that, TRIM25’s degree was 105. Thus, genes such as TRIM25 have an unpredictable potential regarding their interaction relations. As a result, the situations of these types of genes remain unstable, so more time is needed to confirm this information. These types of genes do not have a gene saturation value either.

In conclusion, the gene saturation value depends on the number of related studies but not the gene degree. Meanwhile, although some genes have numerous related studies, their situations remain unstable. These types of genes do not have gene saturation values. Finally, some genes have a high degree but minimal related studies; these types of genes do not exhibit gene saturation. In short, a gene with a high gene saturation value means that this gene has sufficient related studies, and a stable DT curve demonstrating this gene’s interaction partners is generally found.

### Gene saturation in a network with specific types of interactions

**Figure 8:**
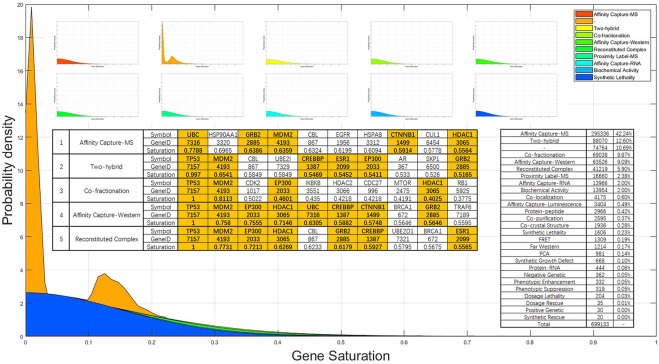
Gene saturation distribution of the top 10 interaction types. Gene saturation distribution of the top 10 types of interaction types. ‘−’ indicates interaction records without a clear label. The table in the figure shows the genes with the top saturation values for the top 5 interaction types. Bold genes with orange backgrounds are the genes with the top 10 gene saturation values in the network of all types of interactions. The table on the right of the figure shows the ratio of the number of different types of interactions to the total number.

There are 26 types of interactions recorded in the data set. These 26 types of interactions can be divided into two categories: genetic interactions and physical interactions. Genetic interactions between two genes mean that mutation/deletion of one gene would have a phenotypic influence on the other gene, such as a positive/negative genetic effect, phenotypic enhancement/suppression, and so on. Physical interactions between two genes means that a physical relationship between the two genes and their products has been demonstrated using a method such as affinity capture, co-localization, protein-RNA, biochemical activity, and so on. Moreover, some gene pairs may have more than one type of interaction. Among all the types of interactions, affinity capture-MS comprises the largest portion at 42.24%. In addition, approximately 10.7% of the interactions lack a clear label. The ratio of the number of different types of interactions to the total number is shown in Fig. 8.

We should note that a gene with high gene saturation does not mean that all the types of interactions of this gene have been studied very well. When researchers study a specific gene interaction network, they may not consider all the types of these interactions. The gene saturation value and order are calculated based on the data of all types of interactions. These values and orders cannot accurately describe the exploration of the stage of genes that only have a specific type of interaction, such as a network with only biochemical activity interactions. Thus, when only a subnetwork with only a few types of interactions is studied, we need to know the saturation of genes with specific types of interactions in the network being studied.

To calculate the gene saturation of each gene in a network with specific types of interactions, we can still use the method mentioned above with minimal differences. We replace the degree with the degree of the gene in the network with specific types of interactions and only consider the portion of related studies that are related to the specific types of interactions. Figure 8 shows the gene saturation distribution of the top 10 types of interactions. ‘−’ indicates interaction records without a clear label. With the exception of interactions without a clear label (the orange area in the figure), each type of interaction has a similar gene saturation distribution. The peak in the gene saturation distribution of interactions without a clear label is closer to 0 compared with the others, which indicates that this portion of interactions has not been studied as much as other types of interactions. The table in Fig. 8 shows the genes with the top saturation values for the top 5 interaction types. The bold genes with orange backgrounds are the genes with the top 10 gene saturation values in the network of all types of interactions.

### Disease Network Saturation

Finally, gene saturation can be used to evaluate whether sufficient research is available to study a gene network. Here, all 84 types of disease gene data for cancer are collected from KEGG (https://www.kegg.jp/kegg-bin/get_htext?htext=br08402_gene.keg) to evaluate whether there are sufficient studies to study these cancer gene networks. To be a network, at least three genes are required. Thus, 64 of 84 cancers are chosen given the presence of more than 3 genes. The average saturation value of all the genes in the disease gene network is used to describe the network’s saturation, and a gene’s saturation value is set to 0 when it does not have a gene saturation value. The average saturation of these 64 gene networks is 0.2859, which is much higher than the average gene saturation of all the genes in gcHGIN, which is 0.02 (the portion of genes that do not have a gene saturation value is also set to 0). This comparison shows that researchers focus on cancer disease genes more than other genes.

Cancers with the top 20 gene network saturations are illustrated in Table 2. All these 20 cancer disease genes are not greater than 10, which can be easily understood given that the fewer genes one disease has, the easier it is to identify all of the interactions between them. In addition, 18 of the top 20 cancers contain disease gene TP53, which has the top gene saturation value, making the average saturation of these cancer gene networks considerably higher than the average gene saturation of all the genes in gcHGIN. However, there are still some cancers with greater than 10 genes that have a large saturation value. Although the saturation values of these cancer gene networks are lower than the top values, these cancers still have higher gene network saturation than the average gene saturation of all the genes in gcHGIN. In addition, this type of disease gene network can have complicated dynamic behaviors compared with networks with only a few genes. In conclusion, most of the cancer disease gene networks have sufficient related studies to make us believe that the unknown interaction relations due to noise in our results are minimal.

**Table 2:** Cancer gene networks with the top 20 saturation values from the KEGG database.

Rank	Disease Name	Gene	Gene Num.	Saturation
1	Burkitt lymphoma	CDKN2A; MYC; TP53	3	0.5536
2	Mantle cell lymphoma	CCND1; CDKN2A; TP53	3	0.5434
3	Adult T-cell leukemia	FAS; CDKN2A; TP53	3	0.5135
4	Hairy-cell leukemia	CCND1; BCL6; TP53	3	0.5092
5	Chronic lymphocytic leukemia	FAS; ATM; BCL2; TP53	4	0.4927
6	Fallopian tube cancer	BRCA1; BRCA2; ERBB2; MYC; TP53	5	0.4525
7	Squamous cell carcinoma	CDKN2A; HRAS; KRAS; TP53	4	0.4388
8	Chronic myeloid leukemia	ABL1; RUNX1; CDKN2A; MECOM; RB1; TP53	6	0.4361
9	Cancer of the anal canal	APC; DCC; TP53	3	0.4289
10	Osteosarcoma	CDKN2A; CDKN2B; MDM2; MYC; RB1; TSPAN31; TP53	7	0.4244
11	Gallbladder cancer	APC; CDKN2A; KRAS; TP53	4	0.4149
12	Endometrial cancer	CTNNB1; ERBB2; KRAS; MLH1; PTEN; TP53	6	0.4065
13	Alveolar rhabdomyosarcoma	ATR; FOXO1; MDM2	3	0.3948
14	Tonsillar cancer	CDKN2A; HIF1A; MYC	3	0.3760
15	Kaposi sarcoma	BCL2; FGF3; KRAS; MYC; TP53	5	0.3722
16	Pancreatic cancer	BRCA2; CDKN2A; ERBB2; KRAS; SMAD4; STK11; TP53	7	0.3653
17	Small cell lung cancer	BCL2; FHIT; MYC; PTEN; RARB; RB1; TP53	7	0.3639
18	Choriocarcinoma	BCL2; CSF1R; EGFR; ERBB2; MDM2; MMP1; MMP2; MYC; TP53	9	0.3520
19	Cholangiocarcinoma	CDKN2A; ERBB2; KRAS; MET; PTGS2; TP53	6	0.3437
20	Oral cancer	CCND1; CDKN2A; EGFR; KRAS; MYC; NRAS; STAT3; TP53	8	0.3368

**Figure 9:**
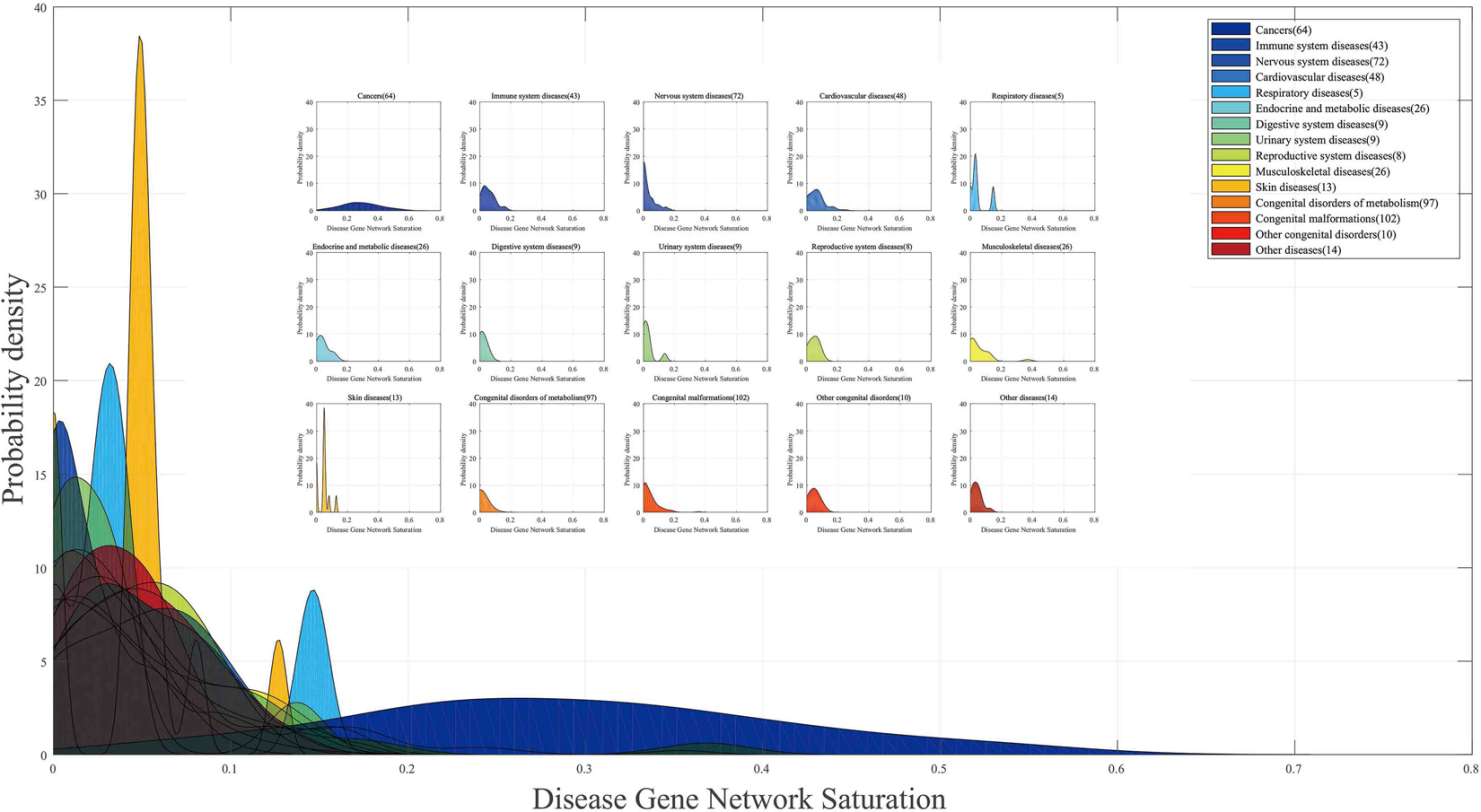
Disease gene network saturation distribution of 15 categories of diseases. There are 546 diseases recorded by KEGG that have more than 3 disease genes. The disease gene network saturation distribution of these 546 diseases, which belong to 15 categories of diseases, is shown.

Furthermore, the saturation values of 546 disease gene networks (gene networks of 546 disease with more than 3 disease genes from all 1728 diseases) that belong to 15 categories of diseases recorded by KEGG (https://www.kegg.jp/kegg-bin/get_htext?htext=br08402_gene.keg) were calculated in this work. The saturation of cancer disease gene networks is significantly higher than that of all the others, which means that the structure of cancer disease gene networks is studied more deeply than other disease gene networks. Although all of cancer disease gene networks’ saturations are higher than the average gene saturation, the gene networks of other categories of diseases recorded by KEGG do not have such high saturation. The gene network saturation distribution of all 15 categories of diseases is illustrated in Fig. 9. Among them, cancer, cardiovascular disease and musculoskeletal diseases are the top 3 categories of diseases with the highest average disease gene network saturations, and congenital disorders of metabolism, digestive system diseases and urinary system diseases are the bottom 3. Disease with higher disease gene network saturation are diseases that are more suitable to study using gene network methods.

## Discussion

In this paper, a method was proposed to evaluate whether there are sufficient related studies on genes to study the network constructed by these genes. As dynamic behaviors in a gene network are significantly affected by the change of the network structure, unknown interaction relations will confound results from gene networks. There are two types of networks. One is the network that was previously known called the *Measured Regulatory Network* (MRN), and the other is the network that really exists in cells called the *Real Regulatory Network* (RRN). Gene networks are mostly constructed by data from the literature and experiments. Thus, a gene’s related literature numbers and degree in gcHGIN are used to calculate an index called gene saturation to describe the distance from MRN to RRN.

The curves of each degree of the gene increase over time (DT curve) have a similar feature: they slowly initially increase, then suddenly rapidly increase, and finally slowly increases. This feature can be fit by the classical Verhulst logistic growth model. Using this model, two key parameters can be obtained: the research focus concentration *r* and the predicted interaction numbers in the RRN *K* of a specific gene. As a feature of each gene, *r* can describe the increasing feature of a specific gene. A gene with a larger *r* means that the gene is more likely to be focused on by researchers over a short period of time. The predicted interaction number in RRN *K* is a key parameter used to calculate the gene saturation. The distance between the degree of a gene in MRN *k* and its predicted interaction number in the RRN *K* can describe the distance from MRN to RRN. However, this *K* may include some noise that affects the result. Thus, the number of related studies needs to be considered at the same time. The larger the amount of related studies, the shorter the distance between MRN to RRN. Our gene saturation satisfied three principles in most cases: the non-decreasing principle, correlation principle and robustness principle. The gene saturation value depends on the number of related studies but not the degree of the gene. Although some genes have a large number of related studies, their situation remains unstable. These types of genes also do not have a gene saturation value. Meanwhile, some genes have a high degree with minimal related studies; these types of genes do not have a gene saturation value. In short, a gene with a high gene saturation value means that it has sufficient related studies, and a stable DT curve proves that this gene’s interaction partners were generally identified. In short, higher gene saturation indicates a reduced increasing rate of the degree of the gene in the future. All genes’ saturation values are calculated in gcHGIN. The gene with the highest gene saturation value is TP53, a tumor related gene called tumor protein p53, with 1239 related studies and a degree of 1051.

Finally, gene saturation has been used to calculate the saturation of disease gene networks. Gene data for all 84 types of cancer from KEGG are used to calculated the saturation of these cancer gene networks, which can evaluate whether sufficient research is available to study these cancer gene networks. The average saturation of these 64 gene networks is 0.2859, which is much higher than the average gene saturation of all the genes in gcHGIN, which has a value of 0.02 (gene saturation is set to 0 for the portion of genes that do not have a gene saturation value). This comparison shows that researchers focus on cancer disease genes more than other genes. As a result, most cancer disease gene networks have sufficient related literature to suggest that only a limited number of unknown interaction relations confound our results. Furthermore, the saturation values of 546 disease gene networks that belong to 15 categories of diseases recorded by KEGG were calculated in this work. The saturation of cancer disease gene networks is significantly higher than that of all the others, which means the structure of cancer disease gene networks is more deeply studied compared with other disease gene networks. Among them, cancers, cardiovascular diseases and musculoskeletal diseases are the top 3 categories of disease with the highest average disease gene network saturation values, and congenital disorders of metabolism, digestive system diseases and urinary system diseases are the bottom 3. Diseases with higher disease gene network saturation are diseases that are more suitable for research using gene network methods.

Gene saturation can provide some advice about which type of gene interaction network is more suitable to study. In the future, the correlation between gene saturation and the increasing rate of the gene degree can be improved by using different ranges of parameters to fit the DT curve or even an improved model. Moreover, these literature data show us the changing process of our MRN from the past until now. If the changing MRN can be obtained, the position of the gene interaction can be predicted. This information may provide some guidance for experimental researchers to choose their research object to improve the efficiency of their research.

## Methods

### Some basic concepts

A gene’s degree is a concept from network science that represents the number of the gene’s interaction partners. In this article, if more than 1 type of gene interaction exists between two genes, the interactions are all counted in the degree of these two genes. The Real Regulatory Networks (RRN) represents the entire interaction network that exists in a real biological system. The Measured Regulatory Network (MRN) represents the known interaction network, which differs based on the exploration of gene interactions. If there is a subnetwork of any gene pairs, there is a path between the two genes. This gene network is called a connected component of the whole network. The giant component of the human gene interaction network (gcHGIN) is the largest connected component of the MRN.

### Function of the fDT curve

Here, the classical Verhulst logistic growth equation is used to obtain the fDT curve as follows:

$${{\mathscr{K}}}_{i}(t)=\frac{{{\mathscr{K}}}_{i}^{0}{K}_{i}}{{{\mathscr{K}}}_{i}^{0}+({K}_{i}-{{\mathscr{K}}}_{i}^{0}){e}^{-rt}},$$

where *k*_*i*_ is a dependent variable that represents the degree of a gene and *t* is an independent variable that represents the time from the first published report of this gene. *K*_*i*_, ${{\mathscr{K}}}_{i}^{0}$, and *r*_*i*_ are three parameters that represent *the maximum degree*, *the initial degree when the first study is published* and *the natural growth rate of gene i*, respectively. We named this fit curve using the classical Verhulst logistic growth model and the increase curve of degree over time (DT increase curve).

This equation is integrated from the following ordinary differential equation:

$$\frac{d{{\mathscr{K}}}_{i}}{dt}={r}_{i}{{\mathscr{K}}}_{i}(\frac{{K}_{i}-{{\mathscr{K}}}_{i}}{{K}_{i}})$$

where the initial value when *t* = 0, ${k}_{i}={k}_{0}^{i}$. This ordinary differential equation can be solved by separation variable method as follows:

$$\frac{d{{\mathscr{K}}}_{i}}{{{\mathscr{K}}}_{i}({K}_{i}-{{\mathscr{K}}}_{i})}=\frac{{r}_{i}}{{K}_{i}}dt$$

Then, we integrated the equation on both sides:

$$\frac{1}{{K}_{i}}\int (\frac{1}{{{\mathscr{K}}}_{i}}+\frac{1}{{K}_{i}-{{\mathscr{K}}}_{i}})d{{\mathscr{K}}}_{i}=\int \frac{{r}_{i}}{{K}_{i}}dt$$

Thus,

$$\frac{{{\mathscr{K}}}_{i}}{{K}_{i}-{{\mathscr{K}}}_{i}}=C{{\rm{e}}}^{{r}_{i}t}$$

Then, this equation can be simplified as follows:

$${{\mathscr{K}}}_{i}=\frac{{K}_{i}}{1+C{{\rm{e}}}^{-{r}_{i}t}}$$

When the initial value is considered, ${{\mathscr{K}}}_{i}\mathrm{(0)}={{\mathscr{K}}}_{i}^{0}$, and we obtain the following:

$$C=\frac{{K}_{i}-{{\mathscr{K}}}_{i}^{0}}{{{\mathscr{K}}}_{i}^{0}}$$

Here, the function of fDT curve can be obtained as described above. Set ${\{{t}_{m}^{i},{k}_{m}^{i}\}}_{m=1}^{{N}_{i}}$ is the degree of gene i when literature *m* is published, where *N*_*i*_ is the number of studies on gene *i*. The least squares method is used to obtain the fDT curve. The parameter vector is Φ = (*K*_*i*_, *r*_*i*_). Parametre vector Φ is the solution of the following optimization problem:

$$mi{n}_{{\rm{\Phi }}}{S}_{i}=\sum _{m=1}^{{N}_{i}}{R}_{m}^{2}({\rm{\Phi }}),$$

where

$${R}_{m}({\rm{\Phi }})={k}_{m}^{i}-{{\mathscr{K}}}_{i}({t}_{m}^{i},{\rm{\Phi }}\mathrm{).}$$

### Calculation of gene saturation

The definition of gene saturation has been mentioned above as

$$\begin{array}{cc}{G}_{s}^{i}=\sqrt{\tfrac{{n}_{i}}{N}\cdot \tfrac{{k}_{i}}{{K}_{i}}} & i=1,2,3\ldots ,{M}_{i}\end{array}$$

where *n*_*i*_ represents the number of interaction-related studies of gene *i*, *k*_*i*_ represents the degree of gene *i* now, *N* represents a standard number of gene interaction studies, *M*_*i*_ is the total number of genes in gcHGIN, and *K*_*i*_ is the solution of the following optimization problem

$$({K}_{i},{r}_{i})={\rm{\Phi }}=arg\mathop{{\rm{\min }}}\limits_{{\rm{\Phi }}}{S}_{i}=\sum _{m=1}^{{N}_{i}}{R}_{m}^{2}({\rm{\Phi }})$$

$$\begin{array}{lll}s\mathrm{.}t\mathrm{.} & {k}_{{N}_{i}}^{i}\le {{\mathscr{K}}}_{i}({t}_{{N}_{i}}^{i})\le \mathrm{3000,} & i=\mathrm{1,}\,\mathrm{2,}\,\mathrm{3,}\ldots ,{M}_{i}\\  & {{\mathscr{K}}}_{i}^{0}=E(\{{k}_{m}^{i}\}), & m=\mathrm{1,}\,\mathrm{2,}\,3\ldots ,\,5\\  & 0\le {r}_{i}\le \mathrm{0.3,} & i=\mathrm{1,}\,\mathrm{2,}\,3\ldots ,{M}_{i}\end{array}.$$

Here, although the gene’s degree *k* is contained in the calculation of gene saturation, the gene saturation is still weakly correlated with the gene’s degree. A larger *k* means a larger *K*, and vice versa. The ratio *k*/*K* is weakly correlated with gene’s degree *K* but is strongly correlated with the growth trends of the gene’s degree.

Regarding genes in the network with only specific types of interactions, we can calculate the gene saturation of these genes by replacing the *n*_*i*_ by the number of the specific types of interaction related literature numbers of gene *i* as ${\tilde{n}}_{i}$, *k*_*i*_ by the degree of gene *i* now in the network with only specific types of interactions as ${\tilde{k}}_{i}$, *N* by a standard literature number of the specific types of interaction-related studies as $\tilde{N}$, *M*_*i*_ by the total number of genes in the gcHGIN of the network with only specific types of interactions as ${\tilde{M}}_{i}$.

$$\begin{array}{cc}{G}_{s}^{i}=\sqrt{\tfrac{{\tilde{n}}_{i}}{\tilde{N}}\cdot \tfrac{{\tilde{k}}_{i}}{{\tilde{K}}_{i}}} & i=1,2,3\ldots ,{\tilde{M}}_{i}\end{array}$$

Therefore, the calculation of ${\tilde{K}}_{i}$ will change the solution of the following optimization problem

$$({\tilde{K}}_{i},{\tilde{\tilde{r}}}_{i})=\tilde{{\rm{\Phi }}}=arg\mathop{{\rm{\min }}}\limits_{\tilde{{\rm{\Phi }}}}{\tilde{S}}_{i}=\sum _{m=1}^{{\tilde{N}}_{i}}{\tilde{R}}_{m}^{2}(\tilde{{\rm{\Phi }}})$$

$$\begin{array}{lll}s\mathrm{.}t\mathrm{.} & \tilde{{k}_{{N}_{i}}^{i}}\le {{\mathscr{K}}}_{i}(\,\tilde{{t}_{{N}_{i}}^{i}})\le \mathrm{3000,} & i=\mathrm{1,}\,\mathrm{2,}\,\mathrm{3,}\ldots ,\tilde{{M}_{i}}\\  & {{\mathscr{K}}}_{i}^{0}=E(\{\,\tilde{{k}_{m}^{i}}\}), & m=\mathrm{1,}\,\mathrm{2,}\,3\ldots ,\,5\\  & 0\le {\tilde{r}}_{i}\le \mathrm{0.3,} & i=\mathrm{1,}\,\mathrm{2,}\,3\ldots ,{M}_{i}\end{array}.$$

### Selection of parameter range and initial value

There are two parameters in model Φ = (*K*_*i*_, *r*_*i*_). For a known gene with maximum degree, such as TRIM25, which has a degree of 2368, it is less possible for genes to have a degree lager than 3000. Meanwhile, a gene’s interaction partners should be larger than the gene degree in gcHGIN. Thus, over the range of *K*_*i*_, *k* is set to 3000, where *k* is the degree of gene in gcHGIN. There may be a sudden increase of the degree of gene. However, this kind of sudden increase does not occur frequently. To avoid these sudden increases in noise in our results, the upper bound of *r* is set to 0.3, which is five-fold greater than the average *r* values of all the genes in gcHGIN. As *r* should be non-negative, the range of *r* is 0 to 0.3. There are three parameters in the model that need an initial value, ${K}_{i}^{0}$,${r}_{i}^{0}$,${{\mathscr{K}}}_{i}^{0}$. The initial value of these parameters is set to ${K}_{i}^{0}=2{k}_{{N}_{i}}$, ${r}_{i}^{0}=0.3$, ${{\mathscr{K}}}_{i}^{0}={\sum }_{m=1}^{5}{k}_{m}$.

### Deletion of part of genes’ saturation

To ensure the high correlation coefficient between the gene saturation and the increase in the gene degree in the near future, there are two parts of a gene whose gene saturation value should be deleted. First, the gene saturation value of those genes with less than 15 related references are deleted. If a sufficient amount of related references is not available, it is difficult to tell whether a majority of this gene’s interactions have been identified. Thus, this part of a gene’s saturation value does not have guiding significance. Second, we deleted the part of a gene’s saturation whose parameter Ki is fixed at upper bound. For this type of gene, the degree generally significantly increases based on recent publications. Given that no subsequent articles are available to provide more unknown interactions about this gene, the exploring stage of this type of gene cannot be assessed accurately, so the saturation of this type of gene is deleted as well. The saturation values of all saturation-deleted genes are set to 0 when the saturation of a network that contains these genes is calculated.
